# Generating local amyloidosis in mice by the subcutaneous injection of human insulin amyloid fibrils

**DOI:** 10.3892/etm.2014.1772

**Published:** 2014-06-11

**Authors:** MARYAM CHINISAZ, AZADEH EBRAHIM-HABIBI, PARICHEHREH YAGHMAEI, KAZEM PARIVAR, AHMAD-REZA DEHPOUR

**Affiliations:** 1 Department of Biology, Science and Research Branch, Islamic Azad University, Tehran, Iran; 2 Biosensor Research Center, Endocrinology and Metabolism Molecular-Cellular Sciences Institute, Tehran University of Medical Sciences, Tehran, Iran; 3 Endocrinology and Metabolism Research Center, Endocrinology and Metabolism Clinical Sciences Institute, Tehran University of Medical Sciences, Tehran, Iran; 4 Department of Pharmacology, Faculty of Medicine, Tehran University of Medical Sciences, Tehran, Iran

**Keywords:** amyloidosis, amyloid, insulin, mouse

## Abstract

Localized deposits of amyloid structures are observed in various pathological conditions. One example of when local amyloidosis occurs is following repeated insulin injections in diabetic patients. The present study aimed to simulate the same condition in mice. To obtain the amyloid structures, regular insulin was incubated at 57°C for 24 h. The subsequently formed amyloid fibrils were analyzed using the Congo red absorbance test, as well as transmission electron microscopy images, and then injected into mice once per day for 21 consecutive days. Firm waxy masses were developed following this period, which were excised, prepared as thin sections and stained with hematoxylin and eosin, Congo red and Sudan black. Histological examination revealed that these masses contained adipose cells and connective tissue, in which amyloid deposition was visible. Thus, localized amyloidosis was obtained by the subcutaneous injection of insulin fibrils. The present results may be of further use in the development of models of amyloid tumors.

## Introduction

Amyloidosis refers to the extracellular accumulation of amyloid fibrils in various tissues and organs, which may result in the disruption of their function. Amyloid fibrils are a specific type of protein aggregate which, upon deposition in particular tissues, may cause serious illness that is often fatal when major organs are involved, or when the amyloidosis is systemic (1,2).

Amyloid tumors, in contrast to systemic amyloidosis, are localized deposits that are usually accompanied by mild clinical symptoms (3). The presence of these deposits has been reported in a number of anatomical sites, including the orbit, neck, oral cavity, breasts, heart, liver and nervous system (3,4). Common clinical conditions associated with amyloid tumors are long-term hemodialysis, chronic inflammation and infections, including tuberculosis and osteomyelitis. Occasionally, patients have been reported to have amyloid deposits in the soft tissue (5), bladder (6) or the respiratory (7) and gastrointestinal tracts (8) without clinical symptoms.

Diabetes mellitus is a highly prevalent illness and a number of diabetic patients require treatment with subcutaneous insulin injections. A previous study demonstrated that insulin injections are associated with local amyloidosis (9). Usually, these are case reports of patients who have detected an abnormal mass in the injection site (10–12). From these studies, it may be hypothesized that amyloidosis may be observed regardless of the location of the injection (13). For example, insulin amyloids have been observed in the shoulders (2), arm (14) and abdominal walls (14–16), and frequently in the areas surrounding the injection site. The formation of insulin fibrils occurs regardless of the source (14) and type (10,11) of insulin administered.

During the course of studying amyloid formation and its prevention, *in vitro* tests on the potential toxicity of amyloid fibrils and associated structures are usually performed on cells (17,18) or organelles (19). The subsequent step to this would be experiments on laboratory animals that are models of the specific amyloid-related disease (for example Alzheimer’s disease) (20–23). However, to the best of our knowledge, an animal model for local amyloidosis has not yet been presented. The method established in the present study may be used as a general representation of local amyloidosis, in a similar manner to the use of the *in vitro* fibril formation of model proteins, as an indicator of the behavior of pathogenic proteins.

## Materials and methods

### Animals

Eight male NMRI mice weighing 26–28 g (average weight 27 g) were obtained from the Pasteur Institute of Tehran (Tehran, Iran) and acclimatized to the new location for a week. All animals were housed under standard conditions with a 12 h dark/light cycle, 50% humidity, a temperature of 22±2°C and free access to water and food (standard pellet feed). The present study was approved by the Animal Ethics Committee of the Science and Research Branch at the Islamic Azad University (Tehran, Iran).

### Amyloid preparation

Regular insulin (EXIR Pharmaceutical Co., Tehran, Iran) was diluted in 50 mM phosphate buffer (pH 7.4) to 0.5 mg/ml. It was incubated at 57°C for 24 h whilst being stirred by Teflon magnetic bars. To confirm the amyloid fibril formation of regular insulin, a Congo red absorbance assay was performed according to a previously described method (24). Images captured under a transmission electron microscopy (TEM; CEM 902A Zeiss microscope; Carl Zeiss, Jena, Germany) were also used as complementary proof. The Congo red kit was obtained from Sigma-Aldrich (St. Louis, MO, USA).

### Experimental groups

Eight mice were randomly divided into two groups (n=4). The first group (control) received daily injections of phosphate buffer (an insulin amyloid vehicle) for 21 consecutive days. The second group (experimental) received daily injections of amyloid fibrils (113 μl) subcutaneously for 21 consecutive days. All groups received their normal diet during the experimental period.

### Histological processing

After 21 days, the waxy masses were excised. Tissue sections were embedded in paraffin and hematoxylin and eosin (H&E), as well as Congo red and Sudan black staining, were applied to each tissue block. A light microscope (Carl Zeiss AG, Oberkochen, Germany) was used to observe the tissue secion.

## Results

### Model development

**Figure 1:**
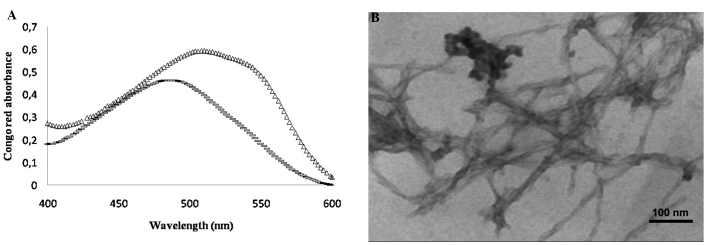
(A) Congo red absorbance spectrum for regular insulin (Δ) after 24 h and Congo red alone (−). (B) Transmission electron microscopy (TEM) image of regular insulin incubated at pH 7.4 and 37°C. The TEM image shows amyloid fibril formed from regular insulin.

The *in vitro* incubation of insulin under amyloidogenic conditions resulted in the formation of insulin fibrils. The shift observed in the absorption spectrum of Congo red (Fig. 1), along with the TEM images indicating the presence of distinct fibrils, were taken as validation of insulin amyloid formation. The pre-formed amyloids were subsequently injected into the mice.

### Tumor formation

After 21 days, no abnormalities in appearance around the injection site were observed in the control group. An abnormal mass was detected in all mice in the experimental group. Two mice from the experimental group were randomly selected, and a biopsy was performed. The masses formed upon amyloid injection were waxy bodies of a white-yellow color and a size of ~10×10×2 mm^3^. The masses appeared similar to the areas of lipohypertrophy observed in human diabetic cases as previously reported (9,25).

### Tissue staining and analysis

**Figure 2:**
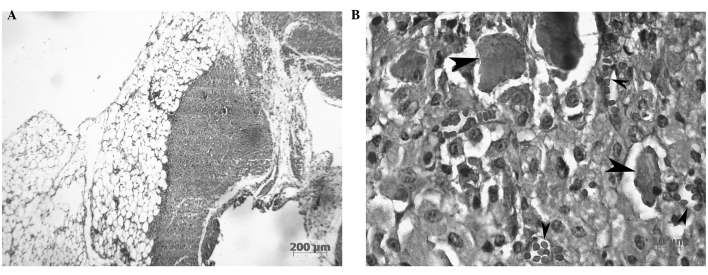
Light microscopic results of excised mass, revealing amorphous eosinophilic material in the hematoxylin and eosin (H&E) staining. Large and small arrows show amyloid deposits and red blood cells, respectively. (A) and (B) are ×10 and ×40 magnifications, respectively.

**Figure 3:**
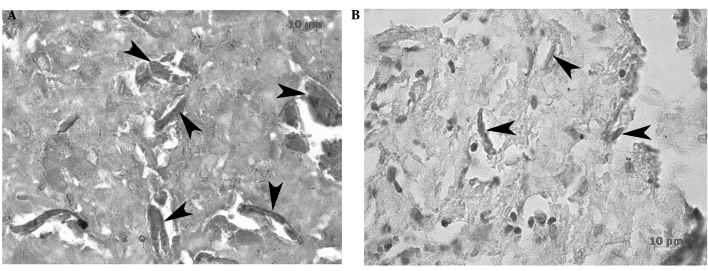
Verification of amyloid deposition in the adipose cells (magnification, ×40). (A) The homogenous depositions are positive with Congo red dye. (B) The adipose cells that surround the amyloid fibril deposition are stained with Sudan black B.

Upon microscopic investigation of the tissues, localized amyloid fibrils surrounded by connective tissue were detected following H&E staining (Fig. 2). Congo red staining was also performed (Fig. 3), since it is a specific stain for the detection of amyloid structures (24). Sudan black staining confirmed that the excised tissue included adipose tissue (Fig. 3). Based on these results, it was concluded that an amyloid tumor containing fibrillar deposits was formed at the injection site of the two mice.

## Discussion

More than 20 proteins and 24 protein precursors that are able to form amyloid fibrils in a comparable manner have been reported (26,27). *In vivo*, the deposition of these fibrils in the extracellular environment results in amyloidosis (28). Usually, soluble proteins become insoluble and are deposited as protein aggregates which then develop into amyloids (29,30). There are various types of amyloidosis which, in general, may be classified as primary (AL) and secondary (AA). AL amyloidosis is related to the deposition of immunoglobulin light chains. AA amyloidosis occurs with chronic disease, especially when an inflammatory process is present (2,12,31). The disease may influence several organs, or may be limited to a particular organ. Symptoms related to amyloidosis depend on the organ involved (32,33).

The skin may become involved with amyloidosis at various levels. Primary cutaneous amyloidosis occurs as nodular, macular and lichen (or papular) amyloidosis (33,34). In the latter two types, amyloid fibrils accumulate in the papillary dermis. An uncommon form of amyloidosis may affect the subcutis, dermis and vascular walls, causing local plasma cell dyscrasia (35,36). Nodular amyloidosis has a higher relapse rate compared with the other forms. The development of local cutaneous disease to a systemic form is rare, but has been reported for nodular amyloidosis (37,38). To the best of our knowledge, no specific therapeutic treatments currently exist for skin amyloidosis and surgical excision is the routine treatment. The method that has been reported in the present study may be expanded and used to test potential treatments for these conditions.

In diabetic patients, a cutaneous amyloid tumor may form at the site of insulin injection (11,25). Within the tumor, lipohypertrophy, which includes the amyloid fibrils, is usually present (11,25). The results of the current study are in accordance with this type of physiopathological finding. From 1983, incidences of amyloidosis in insulin injection sites have been reported in rats (11).

As previously mentioned, there are a limited number of published case reports about cutaneous amyloidosis caused by repeated subcutaneous insulin administration (2,15,14). A limited number of studies have associated cutaneous amyloidosis with non-human insulin, such as porcine insulin, and there are even fewer published studies investigating amyloidosis with human insulin (2,16).

One form of cutaneous amyloidosis is related to local subcutaneous injections of insulin, the incidence rate of which may be underestimated when considering the high prevalence of diabetes mellitus and insulin treatment. The simple method proposed in the present study may be useful in investigating the characteristics of local amyloidosis, as well in the search for potential treatments. The exact mechanism by which insulin-induced amyloidosis occurs remains largely unknown in the scientific literature. Notably, in previous studies, insulin itself was observed to have properties of lipohypertrophy, while the present study used injections of insulin amyloid fibrils and observed the same effect. It has been verified that long-term injection of insulin may result in lipohypertrophy, lipoatrophy and, rarely, infection (25). Further tests are required to ascertain whether insulin amyloid injection may cause the same effects. Another use for the method established in the current study may be to investigate the tendency of various insulin types to cause amyloidosis. Finally, irrespective of the amyloidosis type, the present method may be used to further study local amyloidosis.
